# Neurodevelopmental Disorders and Their Association With Neurodegenerative Disorders: A Systematic Narrative Review

**DOI:** 10.1192/bjo.2024.233

**Published:** 2024-08-01

**Authors:** Rebecca Restorick, Huw Dunstall, Katja Umla-Runge

**Affiliations:** 1 NHS Borders, Selkirk, United Kingdom; 2 Cardiff University, Cardiff, United Kingdom; 3 Swansea Bay UHB, Swansea, United Kingdom

## Abstract

**Aims:**

Neurodevelopmental disorders (NDDs), such as dyslexia, dyspraxia, and dyscalculia affect cognitive function and therefore share symptomology with neurodegenerative disorders, such as Alzheimer's disease, vascular dementia, and frontotemporal lobe dementia. The primary aim of this narrative systematic review is to ascertain if there is an association between NDDs and neurodegenerative disorders. Secondary aims are what the prevalence of NDDs within a dementia population is and what effect these early life learning disorders have on patients as they get older. It was hypothesised that NDDs would overestimate the severity of cognitive impairment, thereby increasing the severity of dementia staging, and impacting patient care.

**Methods:**

Using a Population, Exposure, Comparator, Outcome, Setting, and Study design (PECOS) framework, keywords of “dementia”, “dyslexia”, “Dyspraxia/clumsy child syndrome/developmental apraxia/motor learning difficulty/disorder of attention and motor perception” and “dyscalculia/mathematical learning disability” were searched for on 4 databases (SCOPUS, OVID, Cochrane Central Register of Controlled Trials and Web of Science) from January 1, 1960 – June 10, 2022. Studies were included if they discussed both neurodegenerative and neurodevelopmental disorders or compared an intervention typically used in one disorder on the other (e.g., dementia intervention being used on neurodevelopmental disorder). Studies were excluded from grey literature articles, or if they only discussed a neurodevelopmental or neurodegenerative disorder without reference to the other, or if it included acquired, rather than neurodevelopmental dyslexia, dyscalculia, or dyspraxia.

**Results:**

A total of 8 studies were included for narrative synthesis. The main finding was an association between dyslexia and both Alzheimer's disease and frontotemporal dementia. Many studies suggested this was due to a genetic phenotype that caused a vulnerability in the language regions of patients’ cortices. There was also evidence of structural changes associated with NDDs and increased levels of grey and white matter atrophy in dementia subtypes, particularly in the language areas of the brain.

**Conclusion:**

Due to screening and consequently formal diagnosis of neurodevelopmental disorders only recently coming into education systems, many adults currently attending memory clinics did not have a formal diagnosis. As there was limited research on dyspraxia and dementia, partly due to limited research into dyspraxia itself and without a standardized diagnostic tool for adolescents and adults, further research is needed in this area. The hypothesis of NDDs increasing the severity of dementia staging was also not supported by the literature results, and on the contrary, some studies suggested greater global preservation of cognitive function in patients with NDDs and dementia.

